# Comprehensive proteome analysis of lysosomes reveals the diverse function of macrophages in immune responses

**DOI:** 10.18632/oncotarget.14558

**Published:** 2017-01-08

**Authors:** Yanpan Gao, Yanyu Chen, Shaohua Zhan, Wenhao Zhang, Feng Xiong, Wei Ge

**Affiliations:** ^1^ Department of Immunology, National Key Laboratory of Medical Molecular Biology, Institute of Basic Medical Sciences, Chinese Academy of Medical Sciences, Dongcheng, Beijing, China; ^2^ MOE Key Laboratory of Bioinformatics, School of Life Sciences, Tsinghua University, Beijing, China

**Keywords:** innate immunity, lysosome, macrophage, quantitative proteomics, tandem mass tag labeling, Immunology and Microbiology Section, Immune response, Immunity

## Abstract

Phagocytosis and autophagy in macrophages have been shown to be essential to both innate and adaptive immunity. Lysosomes are the main catabolic subcellular organelles responsible for degradation and recycling of both extracellular and intracellular material, which are the final steps in phagocytosis and autophagy. However, the molecular mechanisms underlying lysosomal functions after infection remain obscure. In this study, we conducted a quantitative proteomics analysis of the changes in constitution and glycosylation of proteins in lysosomes derived from murine RAW 264.7 macrophage cells treated with different types of pathogens comprising examples of bacteria (*Listeria monocytogenes, L. m*), DNA viruses (herpes simplex virus type-1, HSV-1) and RNA viruses (vesicular stomatitis virus, VSV). In total, 3,704 lysosome-related proteins and 300 potential glycosylation sites on 193 proteins were identified. Comparative analysis showed that the aforementioned pathogens induced distinct alterations in the proteome of the lysosome, which is closely associated with the immune functions of macrophages, such as toll-like receptor activation, inflammation and antigen-presentation. The most significant changes in proteins and fluctuations in glycosylation were also determined. Furthermore, Western blot analysis showed that the changes in expression of these proteins were undetectable at the whole cell level. Thus, our study provides unique insights into the function of lysosomes in macrophage activation and immune responses.

## INTRODUCTION

Lysosomes are dynamic vacuolar organelles in which diverse enzymes degrade and recycle extracellular and intracellular materials in an acidic environment. Extracellular material is taken up *via* endocytic or phagocytic pathways, whereas intracellular materials, including a plethora of proteins and damaged organelles, such as mitochondria, are delivered to the lysosomes *via* the autophagic pathway [1]. The lysosomal compartment is of extreme importance for cellular homeostasis and plays a central role in cell clearance, energy metabolism and cell signaling involved in functions such as innate immunity, calcium signaling, or apoptosis [2, 3]. Lysosomal dysfunction results in different types of lysosomal storage diseases (LSD), and the dynamics of lysosomal characteristics during biological and pathological processes has become an area of intense research [3].

Importantly, the structure and function of lysosomes are cell type dependent and regulated by environmental stimuli. The function of lysosomes in immune responses has been recently highlighted, especially the contribution to macrophages in both innate immune and adaptive immune responses. Macrophages are ubiquitously distributed phagocytic cells that ingest and digest foreign materials, dead cells and debris as well as recruiting other immune cells to the infected sites by producing cytokines and chemokines [4]. Lysosomes, which are the destination of pathogens engulfed by macrophages, also play roles in processing and secretion of inflammatory signals [5, 6]. Regarding the adaptive immune system, lysosomal proteolysis generates peptides that bind major histocompatibility complex (MHC) molecules to present crucial information of pathogens for the surveillance by the diverse antigen receptors of the T lymphocyte system [7]. As macrophages change their functions in response to local micro-environmental signals, the roles of lysosomes in these different responses need to be elucidated.

Knowledge of the constituents of an organelle represents the basis of our understanding of its function. Since the discovery of lysosomes nearly 60 years ago, identification of the functional proteins in lysosomes has received significant attention [8]. The majority of hydrolases and cofactors and several lysosomal membrane proteins were characterized as a result of investigations of the mechanisms of LSDs and lysosome-associated disease [9, 10]. In the last decade, the proteomes of the lysosomal matrix and membrane have been extensively studied using large scale mass spectrometry (MS) [11–14]. Most of these studies used lysosomes derived from liver or brain and focused on identifying novel lysosomal proteins; however, knowledge of the proteome of macrophage lysosomes remains fragmentary.

As mentioned previously, macrophages are particularly active phagocytes and also involved in secretion of multiple cytokines. Consequently, macrophage lysosomes are continuously involved in fusion and fission events, which dramatically increase the complexity of their structure and contents [15]. The highly dynamic nature of lysosomes in macrophages due to rapid exchange and communication with other parts of the cell complicates the identification of “true” lysosomal proteins. On the other hand, lysosomal function may also be reflected and regulated by changes in associated proteins originated from other subcellular components. Recognition of exogenous materials in the lysosomal lumen by an array of receptors located on lysosomal membranes results in recruitment of downstream molecules from the cytosol. These molecules are responsible for transferring signals to the nuclei to regulate transcription or to the cell surface for antigen-presentation [7]. In some cases, changes in molecules which are transported to macrophage lysosomes for degradation also indicate the function of macrophages. For instance, macrophages are responsible for the clearance of amyloid β-peptide (Aβ) in the brain, but the dysfunctional macrophages of Alzheimer's disease (AD) patients do not transport Aβ into endosomes and lysosomes [16, 17]. In combination, these observations underline the potential role of the dynamic changes in lysosome-related proteins in reflecting and regulating the function and state of macrophages. Furthermore, lysosomes dynamics is reflected not only in its protein constituents, but also in the modification of these proteins. Proteins, especially membrane proteins localized in lysosomes, are often glycosylated [18]. Glycosylation protects these proteins from proteolysis and is crucial for correct folding and trafficking, thus ensuring their correct functioning in lysosomes [19, 20]. Additionally, Glycosylation at different sites on the same lysosomal protein may be associated with the different functions of this protein [21]. Lysosomes, which are also the center of glycoprotein metabolism, are responsible for degrading and recycling most, if not all, cellular glycoproteins [22]. Identification of new glycoproteins or new glycosylation sites will contribute to our ability to understand and predict their functions. Alterations in glycosylation states may also indicate or contribute to the regulation of lysosomal functions.

The importance of lysosomes for the function of macrophages in immune responses prompted us to investigate the dynamic changes in its proteome in response to infection. In this study, we collected intact and mature lysosomes by a combined process of differential and density centrifugation from RAW 267.4 mouse macrophages infected with bacteria *Listeria monocytogenes (L. m)*, the DNA virus Herpes Simplex Virus 1 (HSV-1) and the RNA virus Vesicular Stomatitis Virus (VSV). These lysosomes were subjected to a large scale tandem mass tag (TMT)-based quantitative MS analysis for profiling of protein abundance and glycosylation stoichiometry. We performed a comprehensive analysis of the changes in proteins that may influence the functions of lysosomes, especially immune-related proteins. Our results provide evidences indicating that distinct events take place in the lysosomal compartment of macrophages according to the type of stimulation, and suggest that lysosomes may play a role in the signaling pathways involved in both innate and adaptive immune responses. Thus, the changes in lysosome-related proteins are indicators not only of the function of macrophage lysosomes, but also of the reactions of macrophages in the face of different pathogenic infections.

## RESULTS

### Isolation of macrophage lysosomes and quantitative proteomic analysis

**Figure 1:**
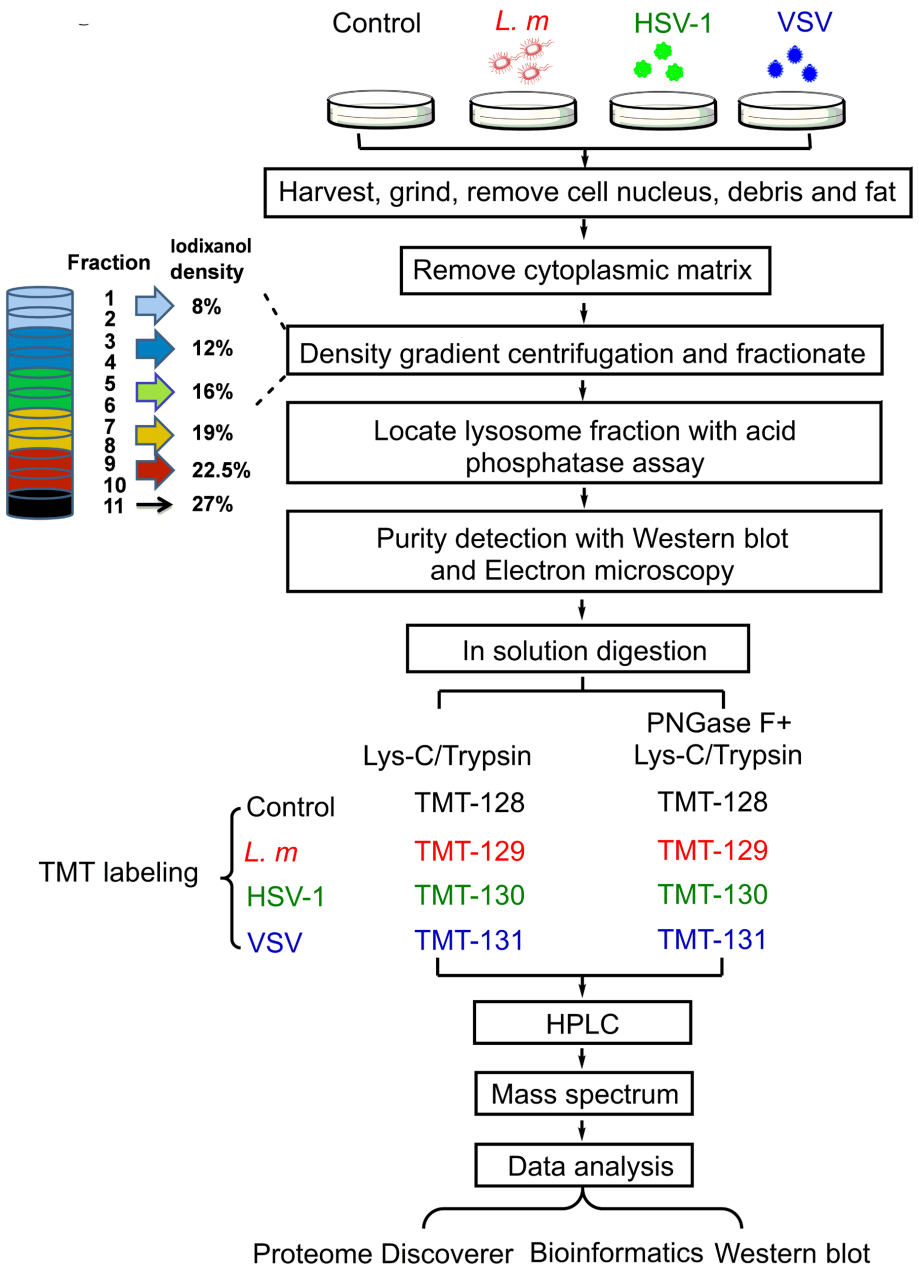
Work flow of the sample preparation for mass spectrometry (MS) analysis Mouse macrophage cell line RAW 264.7 were infected with *L. m*, HSV-1 and VSV or culture medium for 9 hours followed by harvest and purification. Lysosomes were purified by density gradient centrifugation and fractionation. Lysosome purity was detected with western blot and electron microscopy. High purity lysosomes were used in subsequent TMT based quantitative MS and analysis.

To determine the protein composition of lysosomes in infected macrophages, intact lysosomes were collected from the RAW 264.7 murine macrophage cells infected with *L. m*, HSV-1 or VSV as well as the untreated control cells. Lysosome isolation and enrichment was achieved by cell disruption followed by differential centrifugation and density gradient fractionation. These procedures, which allow separation of lysosomes from mitochondria and endoplasmic reticulum (ER), are depicted schematically in Figure 1. Lysosome-enriched fractions were determined by measuring the activity of AP as a marker of the acidic lysosomal lumen [23]. After fractionation, a single peak and similar distribution patterns of AP activity were observed in all groups (Supplementary Figure 1A, 1B). We further validated the distribution of lysosomes by western blot analysis using an antibody for the detection of lysosomal-associated membrane protein 1 (Lamp1), which is considered to be a lysosomal marker protein [24]. In accordance with the AP activity, lysosomes appeared mainly in fractions 1 to 4, with a peaked in fraction 3. The presence of other cell parts in these fractions was also determined. Mitochondria, indicated by the marker proteins cytochrome c oxidase subunit 7b (Cox7b) peaked in fraction 7, with no detectable overlap with the lysosome-containing fractions. Cell plasma membrane, indicated by Ezrin, appeared only in fraction 1. Only a small amount of ER and Golgi apparatus, marked by ERp72 and RCAS1, respectively, was detected in fraction 3. Furthermore, fraction 3 in each experimental group was investigated using electron micrographs. The purity of lysosomes exceeded 80%, with negligible contamination by ER and other organelles (less than 15%) (Supplementary Figure S2), suggesting that the lysosome isolates were of sufficiently high quality for further proteomic analysis.

The enriched lysosomes were digested with Lys-C and trypsin or in combination with peptide-N-glycosidase F (PNGase F), which cleaves between the innermost GlcNAc and asparagine residues, thus generating deamidated asparagine residues that can be discriminated by tandem mass spectrometry. Digested peptides were TMT-labeled for quantification [25]. Labeled peptides were mixed and separated based on their charge and hydrophobicity by mass spectrometry. Peptides were identified by searching a mouse subset of the UniProt database using SEQUEST HT and quantified by evaluation of reporter ion intensities from labels. Searches of the database with a false discovery ratio less than 5% led to the identification of 3,704 lysosome-related proteins. These proteins were observed in all samples, with at least one unique peptide identified. The protein list has been deposited to the ProteomeXchange Consortium [26] *via* the PRIDE partner repository with the dataset identifier PXD002915.

### Molecular characterization of lysosome-related proteins

**Figure 2:**
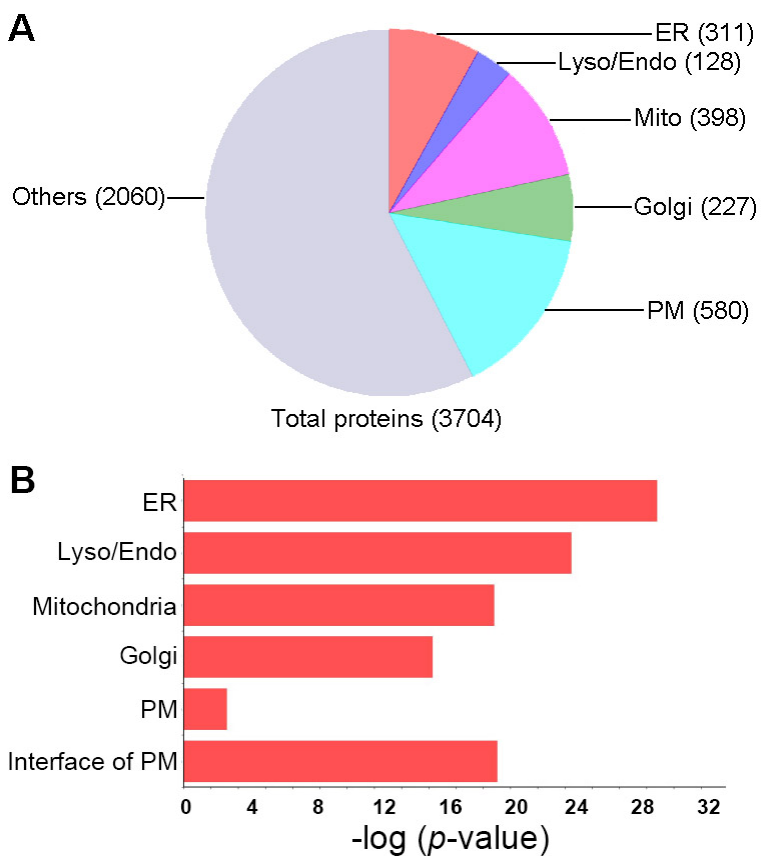
Subcellular distribution of the proteins in lysosomes **A**., Distribution of the proteins identified in lysosome mass spectrometry according to their subcellular annotation. Proteins identified in lysosome were mapped to GO terms of cellular components using DAVID tool, number of proteins in each category was presented. The annotation of proteins localization in other subcellular regions was merged as the “Other” category. ER, endoplasmic reticulum; Mito, mitochondrion; Lyso/Endo, lysosome/endosome; Golgi, Golgi apparatus. **B**., Distribution of the proteins according to the significance of their enrichment in the subcellular localization. P-values were calculated by DAVID tool.

To identify other organelles contributing to these identified proteins, we first characterized the subcellular localization of lysosome-related proteins according to the UniProt database assisted by manual data filtration [27]. Among the 3,704 macrophage lysosome-related proteins identified, 128 proteins were annotated as lysosomal proteins, 311 were attributed to the ER, 398 to mitochondria, 227 to the Golgi apparatus (Golgi) and 580 to the plasma membrane (PM) (Figure 2A). Gene ontology (GO) analysis was performed using the DAVID tools to determine over-represented GO cell component terms [28]. With the exception of the PM, all the intracellular organelle terms mentioned were over-represented (*p*-values approximately 1×10^-25^), indicating active biogenesis of lysosome- and autophagy-related proteins in macrophage cells (Figure 2B).

Interestingly, compared to total PM proteins, proteins attributed to the interface of the PM were more highly over-represented in isolated lysosomes, apparently due to the enrichment of two groups of proteins. The first group comprised the proteins responsible for initiating and regulating endocytosis signals, such as the Rab subfamily members of the Ras GTPase signaling proteins, including, but not limited to, the well-characterized Rab5, Rab7 and Rab11 [29]. The second group consisted mainly of proteins that regulate the generation and trafficking of endosomes, such as clathrin, which is the major component of the endocytic vesicle coat [30], and adaptor protein complexes, which mediate transmembrane protein sorting on the basis of specific peptide recognition [30].

We used TMT-labeled MS/MS approach to determine the changes in the profile of macrophage lysosome-related proteins after pathogen treatment. The abundance of proteins in infected groups was compared to that of their counterparts in untreated group (Control). Distributions of the protein abundance ratios in the three groups displayed a near normal distribution with a mean value near 1, which ensured the fidelity of protein ratios used for subsequent analysis (data not show).

### The abundance of structural proteins in the lysosomal lumen is reduced by infections, while the acidity was unaffected

**Figure 3:**
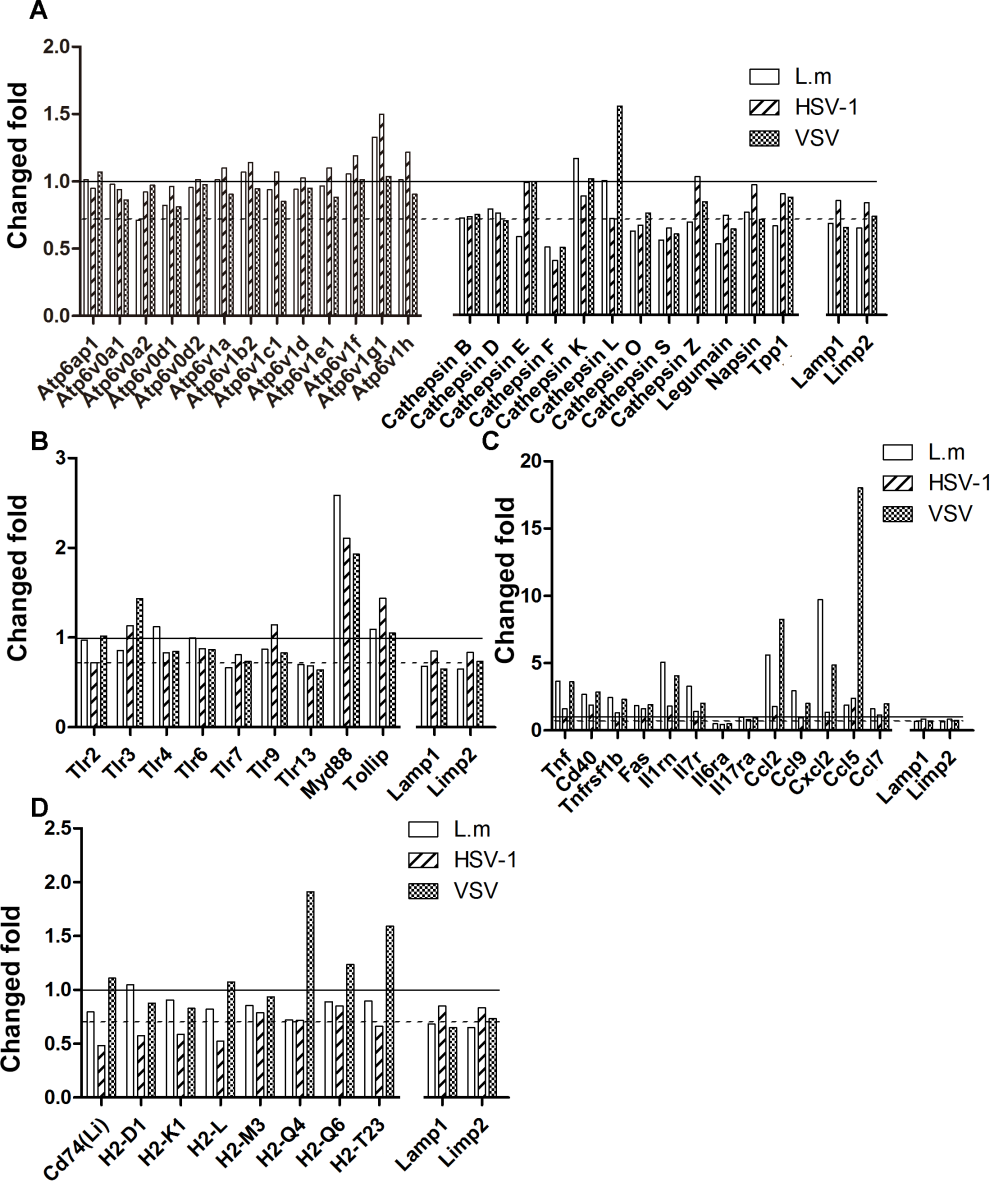
Proteomic alteration of lysosomes reflects the macrophage immune response **A**., The change fold of lysosome specific proteins. The proteins abundances after infections were compared to the uninfected group. Lamp1 and Limp1 are lysosome marker proteins and serves as control. The dashed line equals to the average ratio of Lamp1 and Limp1, which indicates the average ratio of lysosomal structural proteins. The solid line equals to 1. **B**. The change fold of toll-like receptors. **C**. The change fold of inflammatory proteins. **D**. The change fold of MHC molecules.

The response of macrophage lysosomes to infections was first characterized by evaluation of intrinsic lysosomal proteins including major membrane proteins, Lamp1 and Limp2, as well as the repertoire of hydrolases. As shown in Figure 3A, all pathogen treatments decreased the abundance of Lamp1 and Limp2, albeit to different extents, which suggested enhanced lysosomes fusion with other vesicles and the increased rate of lysosomes biogenesis [15]. In a pattern that almost paralleled that of the membrane proteins, the abundance of lysosomal enzymes in the treatment groups decreased compared with that of the control group, with the exception of Cathepsin K and Cathepsin L, the abundance of which remained stable or was even increased after infection with *L. m* or VSV. It is, therefore, conceivable that Cathepsin K and L have independent expression regulation mechanisms. In contrast, lysosomal proton ATPase subunits exhibited an overall unchanged pattern after infection. This observation supports the notion that the acidity of the lysosomal lumen is tightly controlled to allow the arsenal of hydrolases to exert optimal activity.

### Toll-like receptors associated with lysosomes vary according to infection type

Next, we sought to assess the function of the macrophage lysosomes in the immune system. Innate immunity enables the host to develop a rapid and effective response to pathogen invasion and is the first line of defense against infectious agents. Innate immunity is initiated by sensing conserved microbial components termed as pathogen-associated molecular patterns (PAMPs) *via* pattern-recognition receptors (PRRs) including toll-like receptors (TLRs) [31, 32]. In addition to the well-characterized endosomal TLRs (TLR3, TLR7, TLR8, TLR9 and TLR13) [31, 32], we also identified TLR2, TLR4 and TLR6 in macrophage lysosomal fractions (Supplementary Table S1), suggesting their potential functions in sensing the microbial components engulfed by macrophages and also in active communication between the macrophage PM and endomembrane systems.

Microbial components derived from bacteria are mainly recognized by TLRs located on the cell surface [31]. It has been reported that TLR2 is responsible for mediating monocyte activation by *L. m* [33]. However, we did not detect any changes in TLR2 levels in the lysosomal fractions (Figure 3B), which suggested that either the expression of TLR2 was not induced or the recognition of *L. m* components in the lysosomes was accomplished predominantly by other TLRs, such as TLR4. DNA virus HSV-1 infection is sensed by TLR9 molecules, which are located in the endosomal compartment [31, 34]. Our results showed that both TLR3 and TLR9 were increased in the lysosomal compartment, which is consistent with the report that HSV-1 infection results in augmented TLR3 and TLR9 expression [35] (Figure 3B). The increase in TLR3 undoubtedly confirmed its function in defense against HSV-1 [36, 37]. Moreover, the abundance of TLR3 in the lysosomal compartment was also significantly augmented by VSV infection, which was originally supposed to be mediated by the recognition of ssRNA by TLR7 [38, 39].

Following recognition, TLRs undergo conformational changes and initiate downstream signaling cascades to activate NF-κB and induce inflammatory cytokines and type I interferon (IFN) [32, 40]. TLR signaling pathways start with recruitment of different adaptor proteins including myeloid differentiation primary response protein Myd88 or TIR domain-containing adapter molecule 1 (TRIF) [32]. Our results showed that Myd88 located in lysosomes was increased by approximately 2-fold (Figure 3B) after infection with all the pathogen types, suggesting activation of the TLR-Myd88 pathway. Surprisingly, we were not able to detect TRIF, which is used exclusively by TLR3 to induce inflammation, suggesting that activated TLR3 may be involve in other signaling pathways (Figure 3B). Taken together, our results demonstrate that the abundance of proteins associated with macrophage lysosomes, especially the TLRs, varies depending on the pathogen type. Our results reveal that the changes in the lysosomal proteome are indicators of immune pathways activated in macrophages, and suggest that TLR3 plays a role in recognizing both HSV-1 and VSV-1, and Myd88-dependent pathways are activated by all the pathogen types investigated.

### Lysosomes are involved in inflammatory responses with variable function according to the pathogenic stimulation

Lysosomes are also involved in the secretion of inflammation-inducing cytokines and chemokines during immune response [41]. We evaluated the abundance of proteins related to tumor necrosis factor α (TNFα) and interleukin signaling pathways, as well as the chemokine signaling pathways. Overall activation of macrophages was manifested by increase of TNFα and various chemokines as wells receptors for TNFα and interleukins; this is consistent with the observed activation of Myd88 (Figure 3B). However, toll-interacting protein (Tollip), which mediates negative regulation of the TLR-mediated signaling [42], was increased by HSV-1 infection, suggesting that HSV-1 suppresses TLR-induced signaling in inflammatory responses. Indeed, TNF and chemokines were much less abundant compared to the levels in the *L. m* and VSV group, indicating that inflammatory responses are activated less efficiently by HSV-1 (Figure 3C).

### Lysosomal antigen-presentation function is stimulated by VSV

The ER-lysosome system plays a crucial role in adaptive immunity by degrading, processing and loading antigen peptides onto MHC molecules, which are subsequently trafficked to the cell surface for presentation [43, 44]. All the detected MHC-related proteins are also listed in Figure 3D. Taking account of the dilution of lysosomal components, no significant changes in the levels of these proteins were detected following *L. m* infection. HSV-1 infection resulted in decrease of MHC molecules, while MHC I processing proteins were upregulated in the lysosomal compartment, indicating that increased number of MHC molecules were transferred to cell membrane for antigen-presentation. In contrast, VSV infection led to upregulation of both MHC II and MHC I molecules as well as processing proteins including antigen peptide transporter 1/2 (Tap1/2), which suggested overall enhancement of the antigen representation system [45]. It is noteworthy that the expression of Cathepsin L, which that is critical for MHC II invariant chain (Li) processing for antigen loading, was induced only by VSV. This provided further support for the notion that VSV infection augmented the capability of macrophages to present antigens to T cells (44).

### Different defense responses of lysosomes are triggered by different pathogens

**Figure 4:**
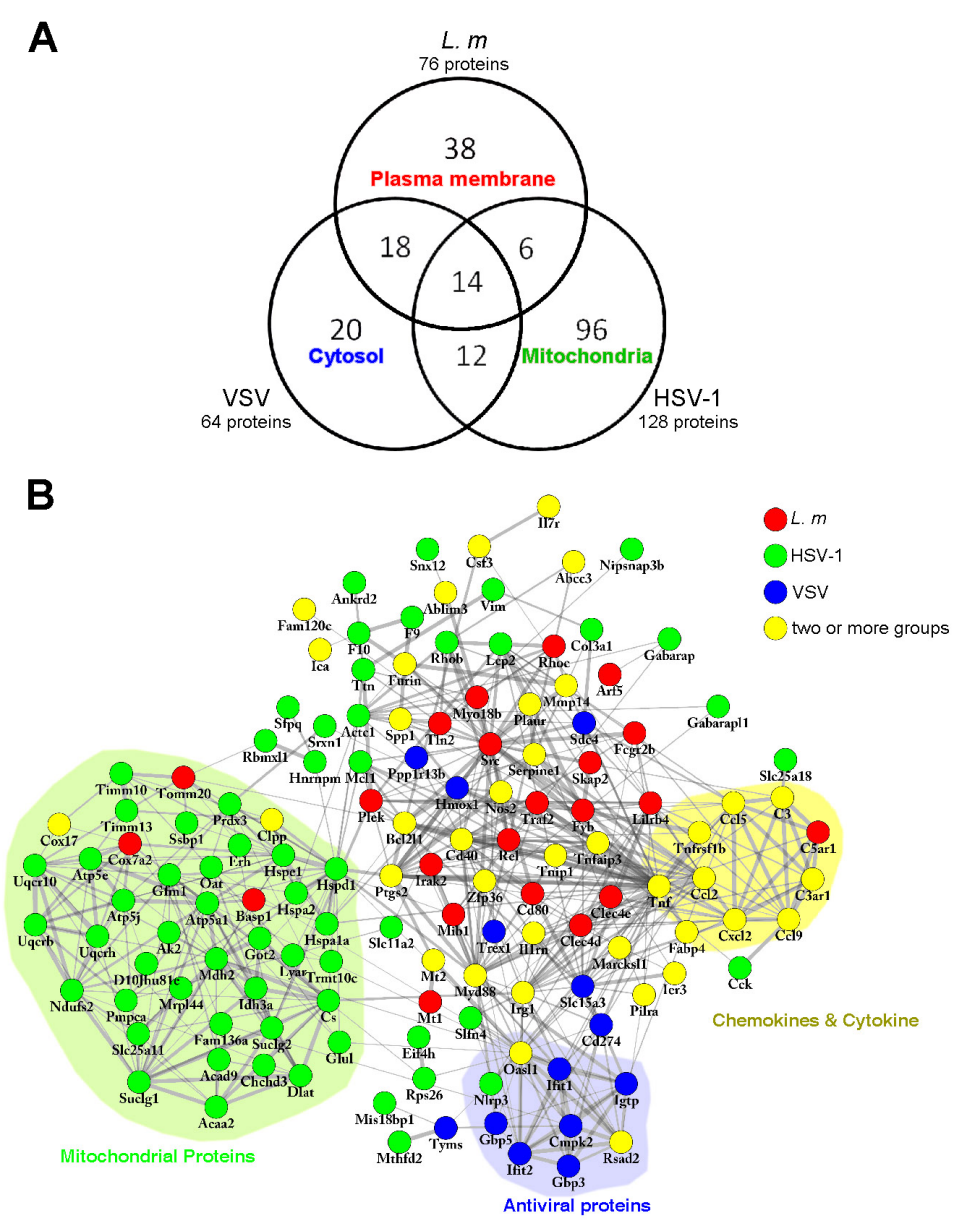
Comparative and network analyses of upregulated proteins in lysosomes **A**., Venn diagram showing the overlap of upregulated proteins (more than 2-fold) between each group. Numbers in each area represents the number of proteins in that category. The descriptions represent the subcellular localizations with highest enrichment in that category. **B**., Upregulated proteins were submitted to STRING for further analysis for protein-protein interaction. Networks were visualized by Cytoscape software. Proteins upregulated by different treatments are indicated by different colors. The proteins of related functions are labeled and indicated by colored backgrounds.

The responses of macrophage lysosomes to infection were determined by examining the proteins that are differentially enriched in lysosomes of pathogen-treated cells compared with untreated control cells. We identified and analyzed 204 increased proteins (02C32-fold compared to the control). Only a minor fraction of these proteins was increased in response to more than one type of infection; thus, each type of infection resulted in a characteristic change in the protein composition of the lysosomes (Figure 4A). The proteins specifically upregulated in response to each type of infection were characterized by GO cellular component analysis. Surprisingly, we found that the proteins upregulated following each type of infection showed distinct distributions among the subcellular regions that were most over-represented. *L. m* infection resulted in an increased abundance of PM components in the lysosomes, while HSV-1 and VSV infections were associated with enrichment of mitochondrial and cytosolic proteins in the lysosomes, respectively.

To investigate the function of the upregulated proteins, we performed functional protein interaction analysis using the STRING database (http://string-db.org) and visualized the network of interaction using the Cytoscape software platform [46]. This generated a network of 137 proteins (nodes) and 500 connections (edges) (Figure 4B). By rearranging the proteins according to the combined scores of connections, three subgroups were identified. Inflammatory factors, such as TNFα and chemokines including Ccl2, Ccl5, Ccl9 and Cxcl2, were upregulated in response to more than one type of infection, particularly *L. m* and VSV (Figure 4B).

The RNA virus, VSV stimulates antiviral activities in macrophages. Interferon-induced protein with tetratricopeptide repeats 1 (Ifit1), a member of the Ifit family that is induced by IFNγ and recognizes viral RNA directly to promote viral clearance, as well as the antiviral proteins Gbp3 and Gbp5, were found to be enriched in lysosomes after VSV infection (Figure 4B) [47, 48].

Surprisingly, numerous proteins attributed to the mitochondria were more enriched in lysosomes by HSV-1 treatment, suggesting that, besides stimulating inflammation and antiviral responses, HSV-1 infection induces mitophagy/macroautophagy activity in macrophages (Figure 4B). Activated mitophagy is important for limiting excess Nlrp3 inflammasome signaling, which is induced and activated by reactive oxygen species (ROS) generated by dysfunctional mitochondria and participates in inflammatory responses and apoptotic cascades [49–51]. Indeed, we detected upregulation of Nlrp3 in HSV-1 infected macrophage lysosomes, indicating disrupted metabolic balance of the mitochondria destined for clearance (Figure 4B).

Compared with the number of upregulated lysosome-related proteins, far fewer were downregulated (02C32-fold), and no significant overrepresentation of GO cell component terms was identified (Supplementary Figure S3A). Functional protein interaction network analysis showed the reduction of Fyn, a member of the Src kinase family that regulates endocytosis in lysosomes in HSV-1 infected macrophages, hinting the endocytosis might be regulated. (Supplementary Figure S3B).

### Identification and characterization of lysosome-related glycoproteins

**Figure 5:**
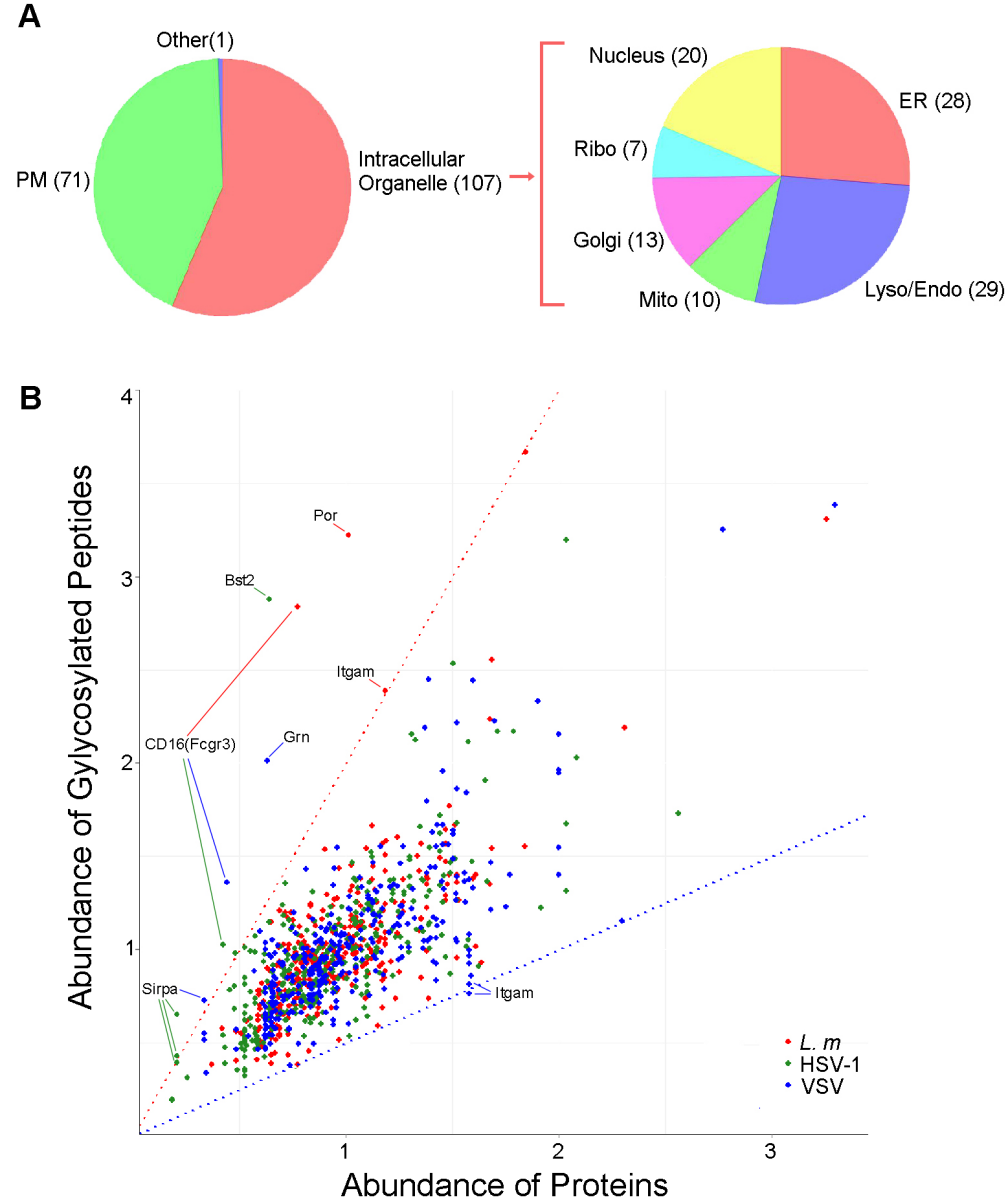
Analysis of lysosome-related glycoproteins **A**., Subcellular distribution of glycoproteins in lysosomes. 193 Lysosome-related glycolproteins identified with mass spectrum were mapped to 179 GO terms of cellular components using DAVID tools, number of proteins in each category was presented. Further classification of intracellular localization is shown. ER, endoplasmic reticulum; Mito, mitochondrion; Lyso/Endo, lysosome/endosome; Golgi, Golgi apparatus, Ribo, ribosome. **B**., Dot plot showing the relative abundance of 300 glycosylated peptides and proteins that each peptide represented; the source of the peptides is indicated by colors. Red and blue dotted lines represent abundance ratios that equal to 2:1 and 1:2, respectively. The peptides that are more glycosylated (with a ratio 02C32:1) and less glycosylated (with a ratio 02C21:2) are labeled.

Glycosylation is one of the most common post-translational modifications of proteins that is known to modulate a variety of biological activities, including folding, trafficking and stability [18]. To investigate the role of glycosylation in the function of lysosomes in response to infection, we sought to identify the glycosylated lysosome-related proteins in macrophages following pathogen treatment by comparison with those in uninfected macrophages. The rationale behind the identification is that the cleavage of N-linked oligosaccharide would leave the deamidated asparagine, which only could be detected after PNGase F treatment. By comparative analysis of peptides digested with or without PNGase F, 300 potential glycosylation sites on 193 proteins were identified. 65 sites have been previously reported and 113 sites have been predicted to be glycosylated by homologue or sequence analysis (Table 1, [71–82] Supplementary Table S2), indicating the feasibility of our approach. Among 122 newly identified potential sites, 59 are located on characterized glycoprotein, and 63 are located on proteins that have never been reported to be glycosylated. Sequence analysis showed that 29 new sites fit with the classic NXT/S glycosylation motif (Table 2). We also sought to identify the consensus motif of the rest of new sites. In detail, the sequences of peptides ± 6 residues flanking the potential glycosylation sites prealigned and analyzed against IPI mouse proteome database with the web-based motif-x tool (http://motif-x.med.harvard.edu/) [52]. However, no motif with significant enrichment was identified (data not shown). The Representative MS/MS data of each category are shown in Supplementary Figure S4. Subcellular localization based on GO analysis showed that most glycosylated proteins were components of intracellular organelles or the PM which is agree with the function of glycosylation. More detailed analysis revealed that the majority of glycosylated proteins were associated with the endomembrane system (Figure 5A).

**Table 1:** Previously reported glycosylation proteins and sites

UniProt-AC	Protein name	Glycosylation sites	reference
P43406	IIntegrin alpha-V; Itgav	N615	Ref. 72
Q75N73	Zinc transporter ZIP14 (Fragment); Slc39a14	N52	Ref. 72
Q61490	CD166 antigen; Alcam	N167, N95	Ref. 72
Q64455	ProteiN-tyrosine-phosphatase; Ptprj	N145	Ref. 73
Q61543	Golgi apparatus protein 1; Glg1	N206	Ref. 73
Q9JIS8	Solute carrier family 12 member 4; Slc12a4	N331	Ref. 72
P97808	FXYD domaiN-containing ion transport regulator 5; Fxyd5	N175	Ref. 72
Q9QUN7	Toll-like receptor; Tlr2	N414, N442	Ref. 74
P97300	Neuroplastin (Fragment); Nptn	N228, N275	Ref. 72
O08912	Polypeptide N-acetylgalactosaminyltransferase 1; Galnt1	N552	Ref. 75
O09117	Synaptophysin-like protein 1; Sypl1	N72	Ref. 72
O88188	Lymphocyte antigen 86; Ly86	N156	Ref. 76
O89001	Carboxypeptidase D; Cpd	N521	Ref. 73
P01901	H-2 class I histocompatibility antigen, K-D alpha chain; H2-K1	N197	Ref. 77
P09055	Integrin beta-1; Itgb1	N669	Ref. 72
P10810	Monocyte differentiation antigen CD14; Cd14	N147, N180	Ref. 78
P11438	Lysosome-associated membrane glycoprotein 1; Lamp1	N97; N177; N252; N259;	Ref. 79; Ref. 72; Ref. 82; Ref. 73
P11688	Integrin alpha-5; Itga5	N596	Ref. 72
P13597	Intercellular adhesion molecule 1; Icam1	N185, N204, N311	Ref. 80
P16675	Lysosomal protective protein; Ctsa	N327	Ref. 82
P17439	Glucosylceramidase; Gba	N289	Ref. 73
P18242	Cathepsin D; Ctsd	N261	Ref. 82
P18572	Basigin; Bsg	N160, N270	Ref. 72, Ref. 73
P24668	CatioN-dependent mannose-6-phosphate receptor; M6pr	N84	Ref. 72, Ref. 73
P31809	Short of Carcinoembryonic antigeN-related cell adhesion molecule 1; Ceacam1	N375	Ref. 73
P40237	CD82 antigen; Cd82	N414	Ref. 72
P41731	CD63 antigen; Cd63	N130	Ref. 73
P46978	Dolichyl-diphosphooligosaccharide-protein glycosyltransferase subunit STT3A; Stt3a	N548	Ref. 73
P58242	Acid sphingomyelinase-like phosphodiesterase 3b; Smpdl3b	N223	Ref. 72, Ref. 73
P70699	Lysosomal alpha-glucosidase; Gaa	N470	Ref. 73
P97449	Aminopeptidase N; Anpep	N784, N817	Ref. 72, Ref. 73
P97797	Tyrosine-protein phosphatase noN-receptor type substrate 1; Sirpa	N246	Ref. 79
Q05769	Prostaglandin G/H synthase 2; Ptgs2	N53	Ref. 80
Q07113	CatioN-independent mannose-6-phosphate receptor; Igf2r	N1532	Ref. 72, Ref. 73
Q3TCN2	Putative phospholipase B-like 2; Plbd2	N520	Ref. 81
Q3TDQ1	Dolichyl-diphosphooligosaccharide-protein glycosyltransferase subunit STT3B; Stt3b	N624	Ref. 73
Q62351	Transferrin receptor protein 1; Tfrc	N725, N730	Ref. 73,
Q8BTJ4	Bis(5'-adenosyl)-triphosphatase enpp4; Enpp4	N279	Ref. 73
Q8C145	Zinc transporter ZIP6; Slc39a6	N68	Ref. 73
Q8C7×2	ER membrane protein complex subunit 1; Emc1	N917	Ref. 73
Q8JZZ7	LatrophiliN-2 (Fragment); Lphn2	N735	Ref. 72, Ref. 73
Q8R2Q8	Bone marrow stromal antigen 2; Bst2	N70	Ref. 73
Q8R5M8	Cell adhesion molecule 1; Cadm1	N104, N116	Ref. 72, Ref. 73
Q91ZX7	Prolow-density lipoprotein receptor-related protein 1; Lrp1	N2128, N3049; N447	Ref. 72; Ref. 73
Q9CPW5	TranslocoN-associated protein subunit beta; Ssr2	N88	Ref. 73, Ref. 82
Q9CQ88	TetraspaniN-31; Tspan31	N117	Ref. 73
Q9CY50	TranslocoN-associated protein subunit alpha; Ssr1	N136, N143	Ref. 72
Q9JKF6	NectiN-1; Pvrl1	N202	Ref. 72, Ref. 73
Q9JKR6	Hypoxia up-regulated protein 1; Hyou1	N515	Ref. 73

**Table 2:** New identified glycosylation protein and glycosylation site with consensus N-glycosylation motif NXS/T

UniProt-AC	Protein name	Glycosylation sites	Remark
Q3TDQ1	Dolichyl-diphosphooligosaccharide-protein glycosyltransferase subunit STT3B; Stt3b	N620	New_site
B2RXS4	PlexiN-B2; Plxnb2	N1005, N530, N761	New_site
Q925Q3	Sodium/potassium/calcium exchanger 6, mitochondrial (Fragment); Slc8b1	N60	New_site
P97300	Neuroplastin (Fragment); Nptn	N196	New_site
P97797	Tyrosine-protein phosphatase noN-receptor type substrate 1; Sirpa	N110	New_site
Q3TB92	AllergiN-1; Milr1	N113	New_site
Q3UP23	Transmembrane protein 26; Tmem26	N102	New_site
Q64735	Complement component receptor 1-like protein; Cr1l	N170	New_site
Q8BXN9	Transmembrane protein 87A; Tmem87a	N62, N127	New_site
Q8R366	Immunoglobulin superfamily member 8; Igsf8	N461	New_site
Q9CYA0	Cysteine-rich with EGF-like domain protein 2; Creld2	N188	New_site
Q9R0E1	ProcollageN-lysine,2-oxoglutarate 5-dioxygenase 3; Plod3	N66	New_site
Q921G6	Leucine-rich repeat and calponin homology domaiN-containing protein 4; Lrch4	N48	New_glycoprotein
O88325	Alpha-N-acetylglucosaminidase; Naglu	N501	New_glycoprotein
P24369	Peptidyl-prolyl cis-trans isomerase B; Ppib	N148	New_glycoprotein
P32883	GTPase KRas	N85	New_glycoprotein
P35980	60S ribosomal protein L18; Rpl18	N40	New_glycoprotein
P54116	Erythrocyte band 7 integral membrane protein; Stom	N135	New_glycoprotein
P58252	Elongation factor 2; Eef2	N21	New_glycoprotein
P61982	14-3-3 protein gamma; Ywhag	N34	New_glycoprotein
Q60766	Immunity-related GTPase family M protein 1; Irgm1	N285	New_glycoprotein
Q8BG07	Phospholipase D4; Pld4	N169, N279	New_glycoprotein
Q8K094	NectiN-2; Pvr	N184, N405	New_glycoprotein
P24452	Capping protein (Actin filament), gelsoliN-like; Capg	N266	New_glycoprotein
Q9CQD8	V-type proton ATPase subunit e 1; Atp6v0e1	N70	New_glycoprotein
Q9R0A0	Peroxisomal membrane protein PEX14; Pex14	N371	New_glycoprotein

Next, we generated a scatter plot of the changes in peptide glycosylation against the changes in glycosylation of the corresponding protein to visualize and evaluate the changes in glycosylation states at each identified site after infection. As shown in Figure 5B, most fluctuations in peptide glycosylation corresponded with the change in the corresponding proteins. From this, it can be inferred that the glycosylation of most proteins remains stable following infection. Nevertheless, the glycosylation level of several peptides was changed. As indicated in Figure 5B Fc gamma receptor III (Fcgr3/CD16), which is a cell surface receptor that is closely associated with phagocytosis and antigen-presentation [53], showed increased glycosylation following infection of any of the pathogen types. In contrast, the glycosylation of another receptor, integrin alpha-M (Itgam or CD11b), which is important for efficient immune-complex clearance and is implicated in several autoimmune diseases [54, 55], was upregulated by *L. m* infection but downregulated by VSV infection. These observations suggested that glycosylation of several proteins is under dynamic regulation and may play roles in mediating immune responses.

### Biological analysis of the state of macrophages by Western blot and immunofluorescence analyses

**Figure 6:**
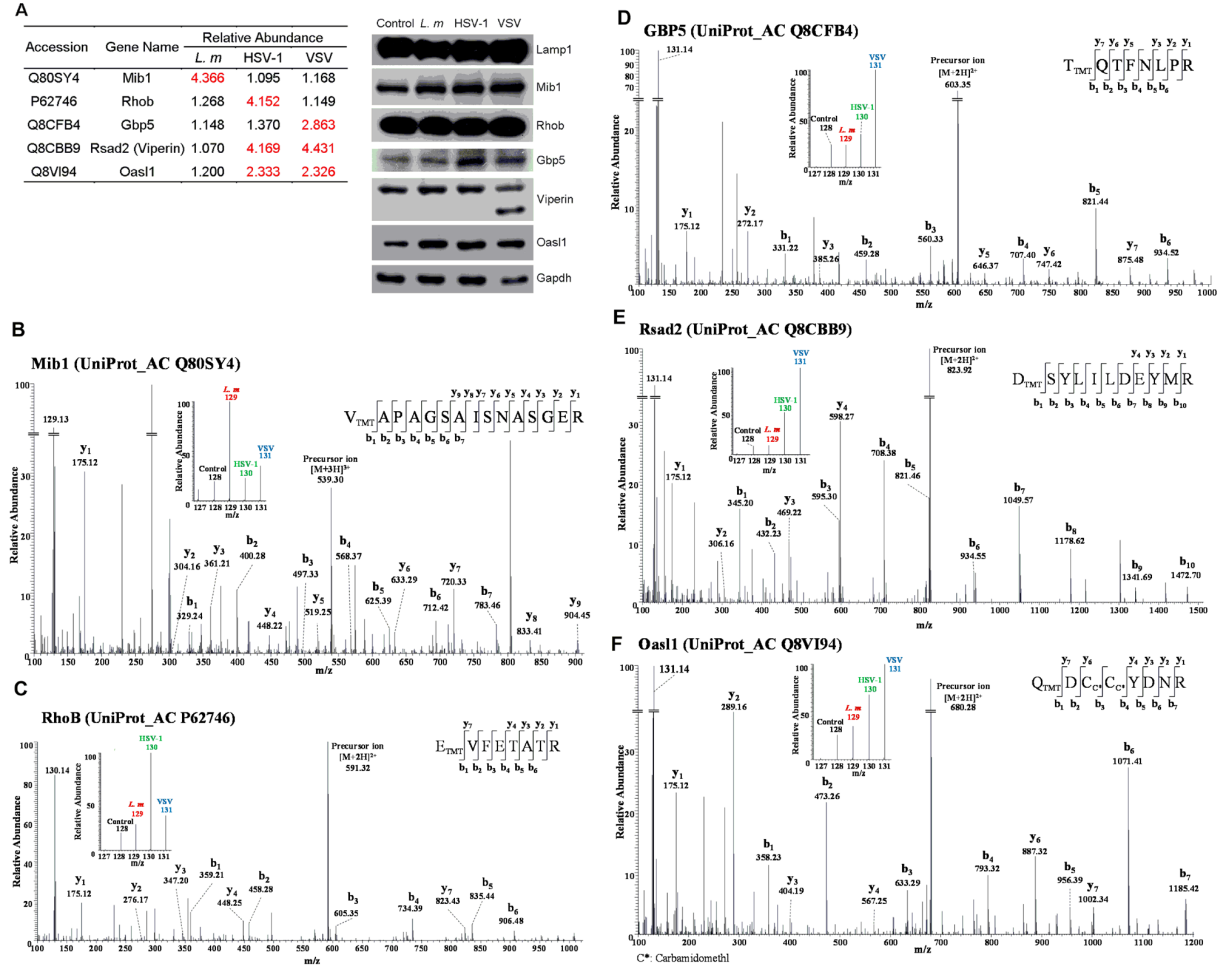
Further analysis of mass spectrometry (MS) results by Western blotting **A**., Immunity-related proteins chosen for Western blot analysis. Relative abundance of 5 proteins quantified by mass spectrum were shown in table. Fold change > 2 is labeled in red (left panel). Right panel, similar amounts of protein extracts from control or infected cells were probed with antibodies against chosen proteins (Mib1, RhoB, Gbp5, Rsad2 and Oasl1). Lamp1 and GAPDH served as loading controls. **B**.-**F**., MS/MS data of represented peptides of chosen proteins. TMT-128 indicates control; TMT-129 indicates *L.* m infection; TMT-130 indicates HSV-1 infection; and TMT-131 indicates VSV infection.

The changes in the abundance of proteins in the lysosomal compartment following pathogen infections may be due to either enhanced expression or recruitment/transport to the lysosomes. To gain further insight, we examined the global expression of several proteins exhibiting different patterns in the MS data. The immunity-related proteins Mib1, RhoB and Gbp5 became more abundant in macrophage lysosomes after treating with *L. m*, HSV-1 or VSV, while lysosomal Oasl2 (Viperin) was augmented by both virus infections (Figure 6A left panel, 6B-6F). However, as shown in Figure 6A right panel, the abundance of these proteins in cells was either unchanged or showed patterns distinct from those in lysosomes, implying that protein profile of lysosomes were not correlated with expression profile. This observation further support the notion that the changes in lysosomes are much more significant and informative compared to that in whole cell, and underlined the specific functions of lysosomes in macrophage activation and immune responses.

**Figure 7:**
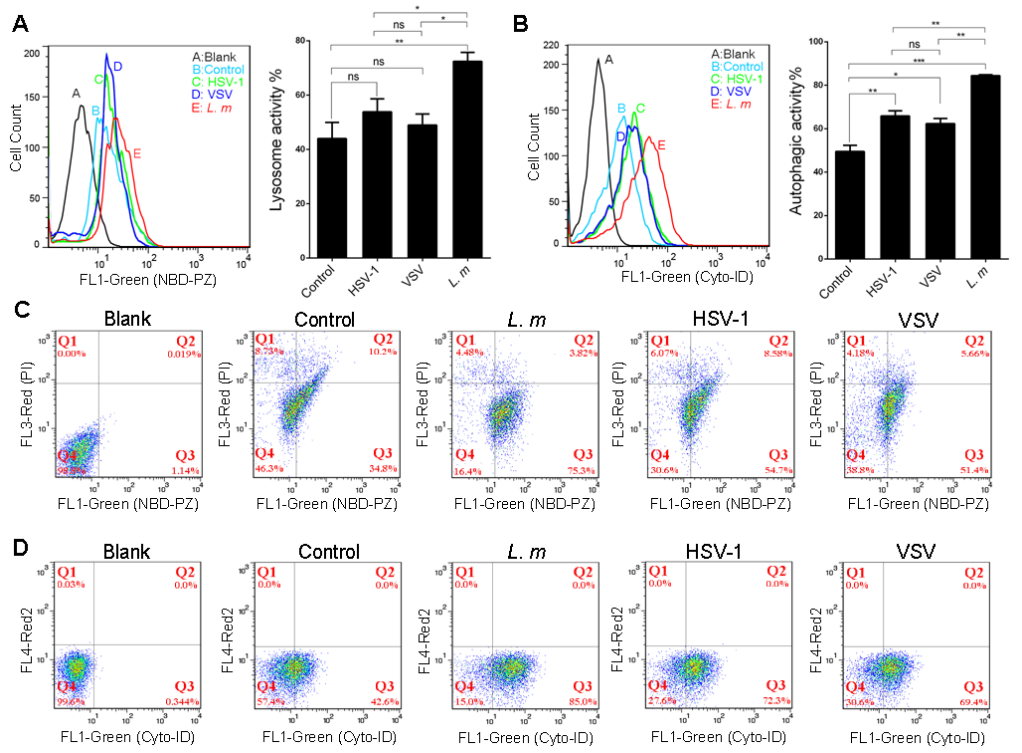
Analysis of lysosome and autophagy activity by cell staining Mouse macrophage cell line RAW 264.7 were infected with *L. m*, HSV-1 and VSV or culture medium for 9 hours. NBD-PZ/PI dual staining was performed for lysosome activity analysis, and Cyto-ID staining was performed for autophagy activity analysis. **A**.-**B**., Quantification of the activity of lysosomes **A**. or autophagy **B**. shows the differences between each group. **C**.-**D**., one of representative image of fluorescence histograms measured using a FACSCalibur showed comparable intensities of lysosome **C**. or autophagy **D**. signals in each group. Cells without staining were set as blank control and the gate was set referring to blank. Mean Values and standard deviations were calculated (*: *p* < 0.05, **: *p* < 0.01, ***: *p* < 0.001, Student's *t*-test).

To corroborate the evidence obtained by HPLC-MS/MS analysis, we evaluated lysosomes function at the cellular level by staining the macrophages with NBD-PZ, which is membrane permeable and reacts with carboxylic acids in the acidic luminal environment of lysosomes. Lysosomal activity was detected by flow cytometry. The results revealed that only *L. m*. infection significantly augmented the function of lysosomes (Figure 7A, 7C). Furthermore, apoptosis/cell death indicated by PI staining was unaffected in all infected cells (data not show). Considering our previous results showing that the ATPase ratio remained stable in lysosomal compartments, we speculate that lysosomes biogenesis is stimulated by *L. m*. Autophagy induction after pathogen infection was determined by Cyto-ID staining. In contrast to lysosome activation, all three types of pathogens stimulated autophagy in macrophages, although the effect of *L. m* was greater than that of HSV-1 and VSV (Figure 7B, 7D)

## DISCUSSION

In this study, we conducted a comprehensive investigation of the protein constituents of macrophage lysosomes and their changes in abundance or glycosylation during macrophage activation by microbial stimulation. We revealed that the changes in the lysosomal proteome varied according to the type of pathogens, and were closely related to the immune functions of macrophages. By comparative analysis, we highlighted the pathways and molecules potentially involved in the pathological responses. Furthermore, the lysosomal proteome changes were not detectable at whole cell level; thus, we conclude that the lysosomes are sensitive indicators of macrophage functions and participate in immune responses.

### Lysosome purification

To obtain a reliable result, our first concern was to ensure the purity of lysosomes, as eliminating signals from co-purified contaminants is critical for subcellular proteomic analysis. Compared to conventional biochemical approaches, modern MS methods provide a much higher dynamic range, sensitivity and accuracy. The lysosomes used in this study were intact and collected by differential and density gradient centrifugation; therefore, our analysis also included the proteins within or attached to lysosomes that are derived from other subcellular compartments.

### The responses of lysosomes to infections

Bacterial pathogens are cleared by phagocytosis and killed by phagosome-lysosome fusion. In accordance with the essential roles of Fc receptors and Src kinases in mediating phagocytosis and phagosome-lysosome fusion, specific upregulation of Fcgr2b and Src were observed in *L. m*-infected macrophages [56]. Unexpectedly, among all the ADP-ribosylation factors (Arfs), which serve as the “signposts” of the vesicles and organizers of membrane traffic in addition to participating in vesicle budding and recruitment of effector proteins [57], only Arf5 showed increased enrichment (02C318-fold) in lysosomes following *L. m* infection (Figure 4B), indicating its importance in phagosome-lysosome fusion. However, the function and biological significance of Arf5 remains to be clarified.

VSV infection stimulated antiviral processes were manifested by the enrichment of Ifit and Gbp family proteins in lysosomes. Recently, there has been increasing interest in Gbp proteins, especially Gbp5. Gbp proteins are induced by IFNγ and restrict pathogen replication by inducing pyroptosis and the secretion of IL-1β/IL-18 [47, 58, 59]. However, although Gbp5 in lysosomes was upregulated by the RNA virus, VSV, only macrophages infected with HSV-1 showed slightly Gbp5 higher expression (Figure 4B). This discrepancy raises the possibility that VSV infection leads to the translocation of Gbp5 to lysosomes for degradation [60].

Our results showed that mitophagy was specifically induced by HSV-1 treatment (Figure 4B). It is known that HSV-1 infection can result in disturbed mitochondrial metabolism and the production of damaging ROS [61], which in turn, stimulate mitophagy to clear the damaged mitochondria [62]. It has been reported that HSV-1 induces clustering of mitochondria around its replication sites in infected cells [63], thus contributing to enhanced mitophagy. Interestingly, after HSV-1 infection, we observed a reduction in amyloid beta precursor protein (App), which is the key protein in the pathogenesis of AD. HSV-1 has long been regarded as a cause of AD; therefore, further investigation of the role of HSV-1 in App degradation is warranted (Supplementary Figure S3B).

### Lysosomes are involved in the activation of IFN pathway after infection

Our proteomic analysis revealed some IFN pathway-related proteins including Rsad2, Oasl1 and Mib1. Rsad2 (Viperin) plays a key role the antiviral response and facilitates production of type I IFN through the TLR7/TLR9 pathway, which is activated by viral nucleic acids [64, 65]. It has been reported that Viperin expression is induced in macrophages by various pathogens including *L. m*. However, our MS analysis showed that Viperin in the macrophage lysosomal compartment was increased by 4-fold after infection with HSV-1 and VSV but not *L. m* (Figure 6A). Furthermore, Western blot analysis showed that VSV infection leads to specific cleavage or degradation of the Viperin protein, which may be important to its biological function.

Similarly, Oasl1 was also induced by all the pathogens, but was only enriched in the lysosomal compartment following HSV-1 and VSV infection (Figure 6A). Interestingly, in contrast to Viperin, which induces the production type I IFN *via* coordination with IRF7, Oasl1 has been reported to negatively regulate the production of type I IFN by inhibiting the translation of IRF7 [65–67].

Mind Bomb1 (Mib1) is an E3 ligase best known for its role in the notch signaling pathway and is responsible for TANK-binding kinase1 (TBK1) ubiquitination. This molecule is critical for production of type I IFN-mediated innate immune responses to RNA viruses. However, its functions in responses to bacteria and DNA viruses are still largely uncharacterized [68–70]. Our results showed that Mib1 was specifically enriched in the lysosomal compartment after *L. m* infection, whereas there were no changes in the expression of Mib1 in macrophages following treatment with the different types of pathogens (Figure 6A).

In combination, these observations demonstrate that lysosomes are involved in type I IFN pathway activation by all types of pathogens in a process that involves recruitment or degradation of certain regulators following infection.

Due to technical limitations and the special functions of lysosomes, we were unable to determine whether the differentially expressed proteins are normally associated with lysosomal functions or are transferred into the lysosomal lumen for degradation and recycling. For instance, the increase of TLR expression could be caused by the increase in receptors localized on the lysosomal membrane for pathogen recognition or by ligand-induced transfer of receptors to lysosomes for recycling or degradation. On the other hand, in both cases, the upregulation of the receptors indicates their roles in recognizing certain type of pathogens. Thus, although our results demonstrate the involvement of these proteins and lysosomes in immune responses, the causes and effects of these changes may vary and further investigations are required to clarify these issues.

In summary, we provide a comprehensive profile of the lysosome-related proteome and glycoproteome. By investigating the dynamic changes in the macrophage lysosome-related proteome during pathogen infection, we demonstrate the involvement of lysosomes in macrophage immune response regulation *via* various pathways, some of which are still incompletely understood. This study extends the previous knowledge of lysosomes function in immunity and provides a basis for further investigations of macrophage immune responses as well as potential drug development targets.

## MATERIALS AND METHODS

### Experimental design

The purpose of this study was to comprehensively understand the role of macrophage lysosomes in the face of different pathogens. Our design used the mouse macrophage RAW 264.7 cell line as a model and included one control and three different pathogens (*L. m*, HSV-1 and VSV) under treatment conditions. The procedure workflow is shown in Figure 1. Lysosomes purified from four conditions were homogenized for subsequent analysis. Lysate from each group was divided into two equal amount samples. One sample was prepared for Lys-C and trypsin digestion for peptide/protein identification; the other was prepared for Lys-C and trypsin followed by PNGase F digestion for peptide/protein identification as well as glycosylated site screening. After labeled with TMT 6-plex, each group was mixed as 1:1:1:1 and subsequently applying HPLC and MS/MS experiment. After data analysis, we obtained two search results: one is treated with Trypsin & Lys-C only result (named MS140038-N), the other is treated with PNGase F followed with Trypsin & Lys-C digestion (named MS140038-G).

### Cell culture

RAW 264.7 cells were cultured in Dulbecco's modified Eagle's medium (DMEM) supplemented with 10% fetal bovine serum (FBS). *L. m* was cultivated in Brain Heart Infusion (BHI) and HSV-1 and VSV were amplified in VERO cells as described previously [83]. RAW 264.7 cells were infected with *L. m*, HSV-1 or VSV at a multiplicity of infection (MOI) of 10. Cells were harvested 9 h post-infection.

### Lysosomes purification

Lysosomes were purified with a Lysosome Isolation Kit (LYSISO1) with slight changes to the manufacturer's instructions. Cells were washed twice with PBS and centrifuged at 800 ×g for 10 min. The pellet was suspended in extraction buffer and homogenized with a 15 mL Dounce homogenizer (approximately 30 cycles). The degree of cell rupture was assessed under an optical microscope. After ultracentrifugation (150,000 ×g for 4 h), samples were fractionated from top to bottom into 11 fractions. The location of the lysosomal fractions was verified using an acid phosphatase (AP) assay kit. Protein concentration was measured with a BCA protein assay kit. The lysosomes purity was detected with electron microscopy. Electron microscopy images were taken by a JEOL TEM1400 transmission electron microscope.

### Tandem mass tag labeling

Lysosomes were dissolved in 8 M urea/PBS lysis buffer containing proteinase inhibitors followed by protein concentration measurement using a Nano Drop 2000. Equal amounts of protein (100 µg) from each group were treated with 10 mM DTT at room temperature for 1 h followed by 25 mM IAA at 50°C for 1 h in the dark. Subsequently, the reaction solution was diluted with PBS to give a final urea concentration of 1 M. For peptide/protein identification, Lys-C was added at a protein/protease mass ratio of 100: 1 and Trypsin was added at a ratio of 25: 1 sequentially. Both enzymatic digestion processes were incubated overnight at 37°C. For glycosylation screening, samples were incubated with PNGase F at the concentration of 4 units per 100 µg protein for 3 h at 37°C before Lys-C and trypsin digestion. The experimental procedure for TMT isobaric labeling was performed according to the manufacturer's instructions with a few modifications. Briefly, samples were desalted with Oasis^®^ HLB extraction cartridges, and labeled with 0.8 mg TMT 6-plex reagent. Peptides derived from control, *L. m*, HSV-1 and VSV were labeled with TMT-6 plex-128, TMT-6 plex-129, TMT-6 plex-130 and TMT-6 plex-131, respectively. Equal amounts of labeled peptides from the four groups were mixed and desalted. The product was dried and dissolved in 100 µl 0.1% trifluoroacetic acid (TFA) for subsequent high performance liquid chromatography (HPLC) separation.

### High performance liquid chromatography (HPLC)

The peptides were separated with the UltiMate 3000 UHPLC (Thermo scientific). The total combined TMT-labeled sample was loaded onto a C18 peptide separation column (Xbridge BEH300, C18, 5 µm, 4.6×250 mm, Waters). Gradient elution with water/acetonitrile solution at pH 10.0 was used for fractionation of the peptides into 50 microtubes (1.5 mL liquid per tube). The fractions were dried and combined to give 20 samples based on peptide concentration and then dissolved in 20 µl 0.1% TFA for further liquid chromatography (LC)-MS/MS analysis.

### LC-MS/MS

The peptide mixtures were separated using the UltiMate 3000 RSLCnano System (Thermo) equipped with an Acclaim PepMap RSLC column (75 µm, 150 mm, Upchurch) custom packed with C18 resin (300 Å, 5 µm) with a 60 min gradient elution at a flow rate of 0.3 µL/mL followed by online electrospray ionization for MS and tandem mass spectrometry (MS/MS) analyses in the Q Exactive Hybrid Quadrupole-Orbitrap Mass Spectrometer (Thermo) with ionic voltage +2.3 kVa. Full scan masses were measured at a resolution of 70,000 full width at half maximum (FWHM) in the orbitrap. The top 20 most abundant precursor ions were sent to a higher energy collision induced dissociation (HCD) cell for fragmentation (MS/MS) with 27% normalized collision energy and fragments were submitted to the orbitrap mass analyzer for data collection. The MS/MS spectra were acquired at a resolution of 17,500 FWHM.

The mass spectrometry proteomics data have been deposited to the ProteomeXchange Consortium [26] *via* the PRIDE partner repository with the dataset identifier PXD002915.

### Database searching and criteria for protein identification

MS data analysis was analyzed with Proteome Discoverer 1.4 (Thermo Scientific). In detail, SEQUEST HT algorithm was used to match MS data files with the UniProt/SwissProt mouse database (released on June 15^th^ 2015, 53185 entries). Mass spectra was filtered based on minimum number of peaks of 200 and intensity of 20,000 before submitting to SEQUEST HT. Full tryptic activity was applied as enzymatic pattern with a maximum of two missed cleavage sites, a minimum 6 peptides and a maximum 144 peptides length. The search algorithm was set as the following parameters: precursor tolerance, 20 ppm; fragment tolerance, 0.02 Da; static modification, cysteine carbamidomethylation (+57.021) and lysine and N-terminal TMT 6-plex modification (+229.163). Methionine oxidation (+15.995) and asparagine and glutamine deamination (+0.984) were specified as dynamic modifications. Protein identification was considered valid if at least one peptide was statistically significant (*p* < 0.05 with a false discovery rate (FDR) at 5% determined by Proteome Discoverer 1.4).

Deamidated sites appeared only in PNGase F treated group were defined as glycosylated sites.

Protein quantification was performed with processing node reporter ions quantifier using the TMT 6-plex method. Integration tolerance was set as 20 ppm. All peptides ratios were normalized to the median protein ratio. Proteins were quantified with unique peptides. The differential expression threshold was defined as 2-fold change.

### Cyto-id^®^ autophagy detection dye staining and lysosomes/cytotoxicity dual staining

RAW 264.7 cells were seeded in 6-well plate at a density of 5×10^5^ cells per well. After 8 h culture, cells were infected with *L. m*, HSV-1 and VSV at MOI = 10 for 9 h. For Cyto-ID^®^ Autophagy Detection Dye Staining, cells were treated with 0.5 µM Rapamycin for 16 h as positive control. Cells were harvest and treated with Cyto-ID^®^ Autophagy Green dye Staining Solution at 37°C for 30 min in the dark. For Lysosomes/Cytotoxicity Dual staining, cells were treated with 25 mM Chloroquine for 12 h as positive controls. Cells were harvest and treated with NBD-PZ/Propidium Iodide Dual Staining Solution at 37°C for 10 min in the dark. Signals were detected by Millipore guava^®^ Flow Cytometry easyCyte6-2L system (Billerica, Massachusetts, USA). Data analysis was carried out with FlowJo software Version 7. RAW 264.7 without treat and staining signals were blank and the gate was set referring to blank.

### Western blot analysis and antibodies

Whole cell lysates prepared from infected or control RAW 264.7 were subjected to Western blot analysis. Proteins selected based on the MS data were probed using the specific antibodies as indicated: Cox7b (ab140629), Oasl1 (ab116220), Mib1 (ab124929), Viperin (ab73864), Lamp1 (ab25245), Gbp5 (sc-160354) and GAPDH (M171-3). ERp72 (cst-5053), RCAS1 (cst-12290), Ezrin (ab75840).

### Statistical analysis

For functional pathway analysis, the different subsets of proteins identified in the data set were subjected to functional analysis using DAVID bioinformatics resources (28). Gene ontology terms for biological processes (BP), molecular functions (MF) charts and cellular components (CC) were obtained using default statistical parameters (threshold: count 2, ease 0.1).

STRING (Search Tool for the Retrieval of Interacting Genes/Proteins) database (http://string-db.org) was used for predicting the protein networks. All STRING network analyses were performed using protein accessions as input and with the following parameters: “Experimental” and “Databases” evidences at medium (0.4) confidence level. For visualization, STRING networks were exported to PSI-XML and imported into Cytoscape.

The results are presented as mean ± S.D. values from at least three experiments, and statistical analyses were performed by either paired/unpaired Student's *t*-test or one-way ANOVA. The statistical significance of differences was set at *p* < 0.05 (*) or *p* < 0.01 (**) or *p* < 0.001 (***).

## SUPPLEMENTARY MATERIALS FIGURES AND TABLES







## Abbreviations

L. m: Listeria monocytogenes
HSV-1: herpes simplex virus type-1
VSV: vesicular stomatitis virus
LSD: lysosomal storage diseases
TMT: tandem mass tags
Aβ: amyloid β-peptide
AD: Alzheimer's disease
MOI: multiplicity of infection
AP: acid phosphatase
FDR: false discovery rate
ER: Endoplasmic reticulum
PM: plasma membrane
Cox7b: cytochrome c oxidase subunit 7b
PNGase F: peptide-N-glycosidase F
Limp2: lysosome membrane protein 2
Lamp1: lysosomal-associated membrane protein 1
PAMP: pathogen-associated molecular patterns
PRR: pattern-recognition receptor
MHC: major histocompatibility complex
Tollip: toll-interacting protein
ROS: reactive oxygen species
Nlrp3: NACHT, LRR and PYD domains-containing protein 3
Arfs: ADP-ribosylation factors
TBK1: TANK-binding kinase1
Rab: ras-related protein Rab
TLR: toll-like receptor
Myd88: myeloid differentiation primary response protein MyD88
TRIF: TIR domain-containing adapter molecule 1
TNF: tumor necrosis factor
IFN: interferon
Tap: antigen peptide transporter
Ifit: interferon-induced protein with tetratricopeptide
Fcgr: Fc gamma receptor
Itgam: integrin alpha-M
Oasl2: 2’-5’-oligoadenylate synthase-like protein 2
Mib1: E3 ubiquitin-protein ligase MIB1
RhoB: Rho-related GTP-binding protein RhoB
App: amyloid beta precursor protein
GO: gene ontology
