# Supplementary material for: Comprehensive proteome analysis of lysosomes reveals the diverse function of macrophages in immune responses
Source: Oncotarget. 2017 Jan 8;8(5):7420–40. doi: 10.18632/oncotarget.14558 (PMC5352332; doi:10.18632/oncotarget.14558)
Supplement: Supplementary file 1 [file oncotarget-08-7420-s001.pdf]

Comprehensive proteome analysis of lysosomes reveals the diverse function of macrophages in immune responses

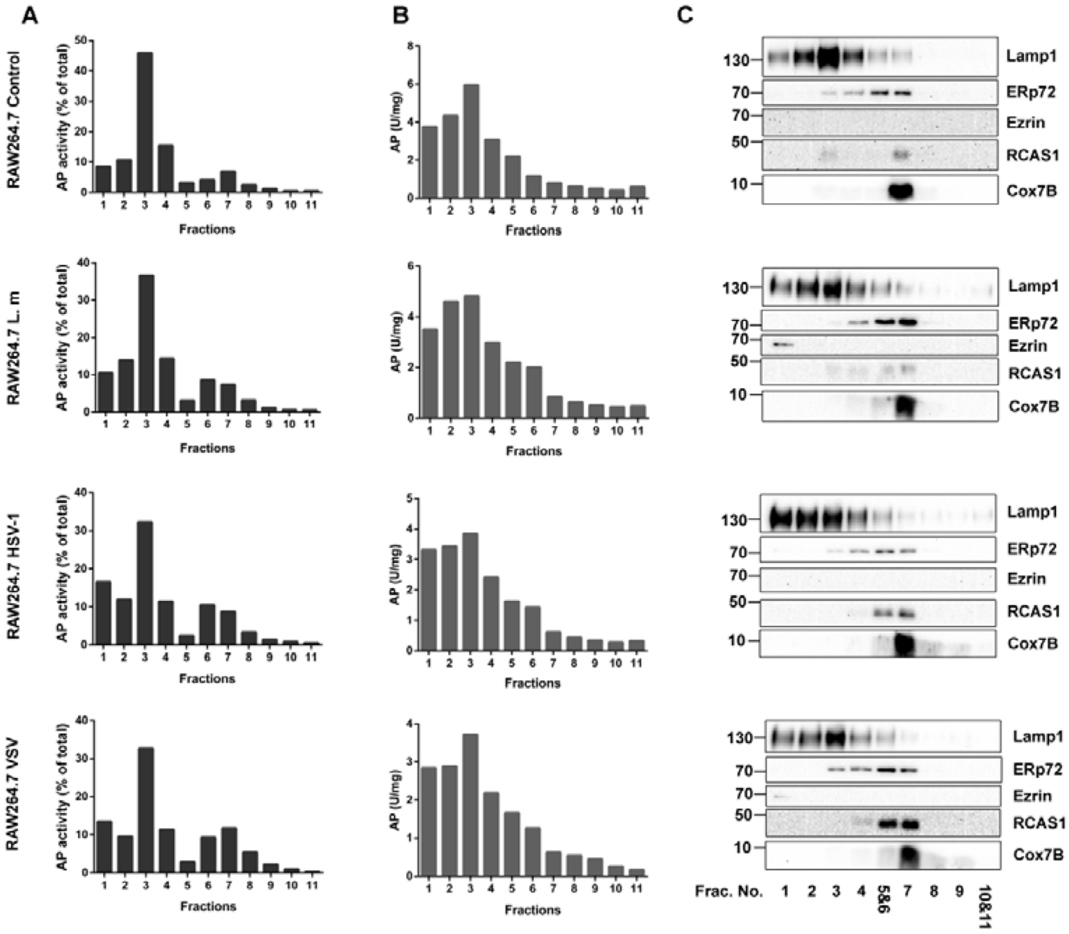

Supplementary Figure 1: Analysis of density Gradient fractions. Mouse macrophage RAW 264.7 cells were harvested after infected with *L. m*, HSV-1 and VSV or culture medium for 9 hours. Subcellular organelles were separated using density gradient centrifuge and total 11 fractions were collected for subsequent analysis. A, The distribution of AP in each fractions. B, The AP normalized to protein concentration in each fraction. C. The presence of lysosome (Lamp1), endoplasm reticulum (ERp72), plasma membrane (Ezrin), Golgi apparatus (RCAS1) and mitochondria (Cox7B) was determined by western blot using antibodies against their marker proteins. 15 ug proteins were loaded in each lane. Fraction 5-6 and 10-11 were pooled, respectively, due to their low protein concentration.

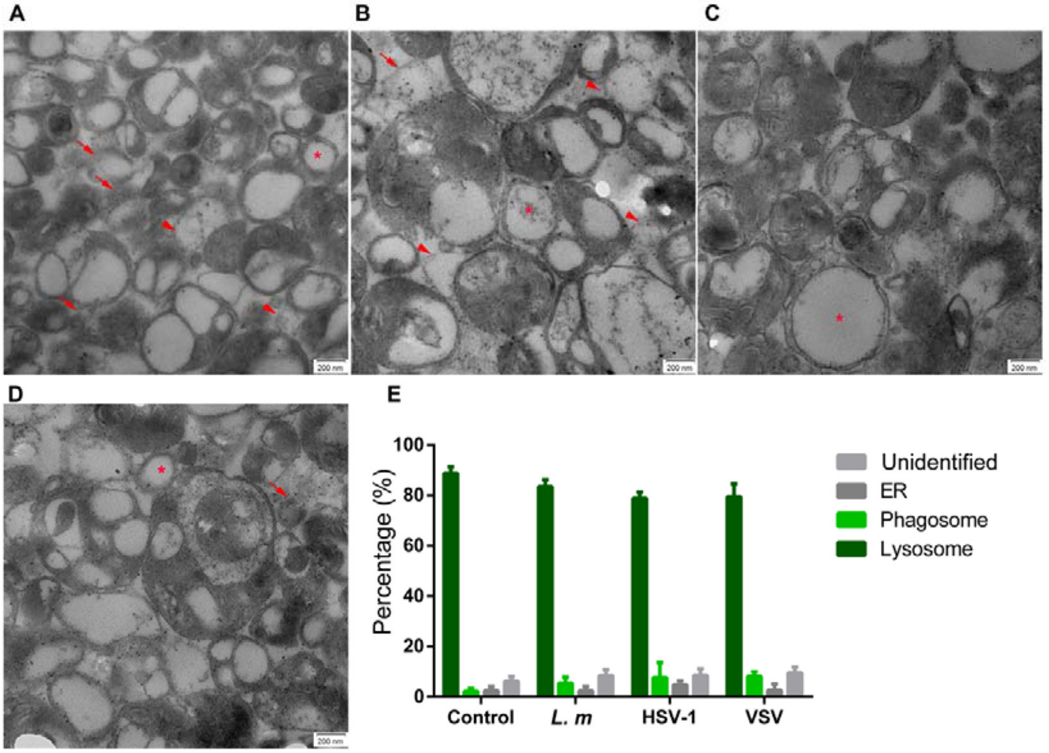

Supplementary Figure 2: Verification of the purity of lysosomes by electron microscopy. A-D, Representative electron microscope images (40,000×) of enriched lysosomes derived from the control (A) or macrophages treated with *L.m* (B), HSV-1 (C) or VSV (D). Red asterisk indicates phagosomes; red triangle indicates ER; and red arrow indicates unidentified organelles. E, Statistic of the organelle composition of fraction 3 in each group.

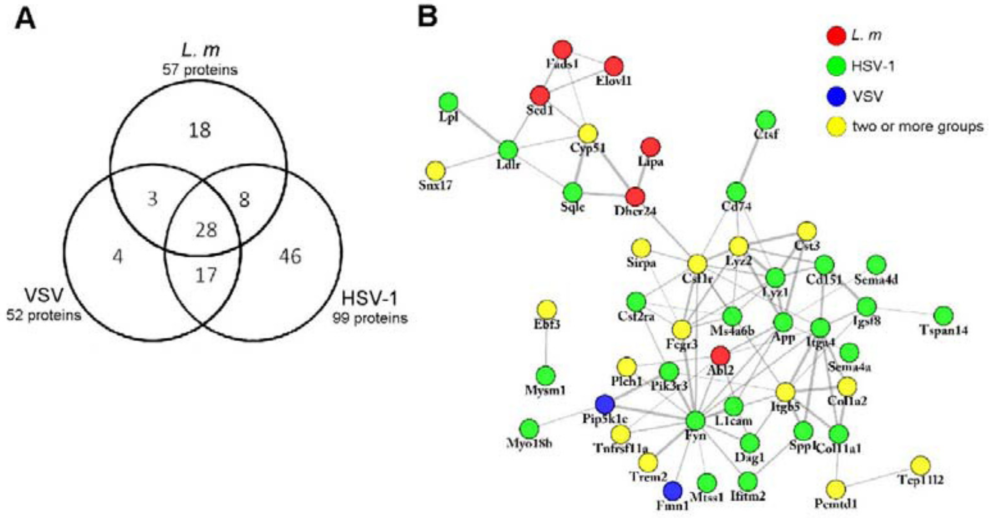

Supplementary Figure 3: Comparative and network analyses of down-regulated proteins in lysosomes. A, Venn diagram showing the overlap of downregulated proteins (more than 2-fold) between each group. Numbers in each area represents the number of proteins in that category. B, Downregulated proteins were submitted to STRING for further analysis for protein-protein interaction. Networks were visualized by Cytoscape software. Proteins downregulated in different groups are indicated by different colors.

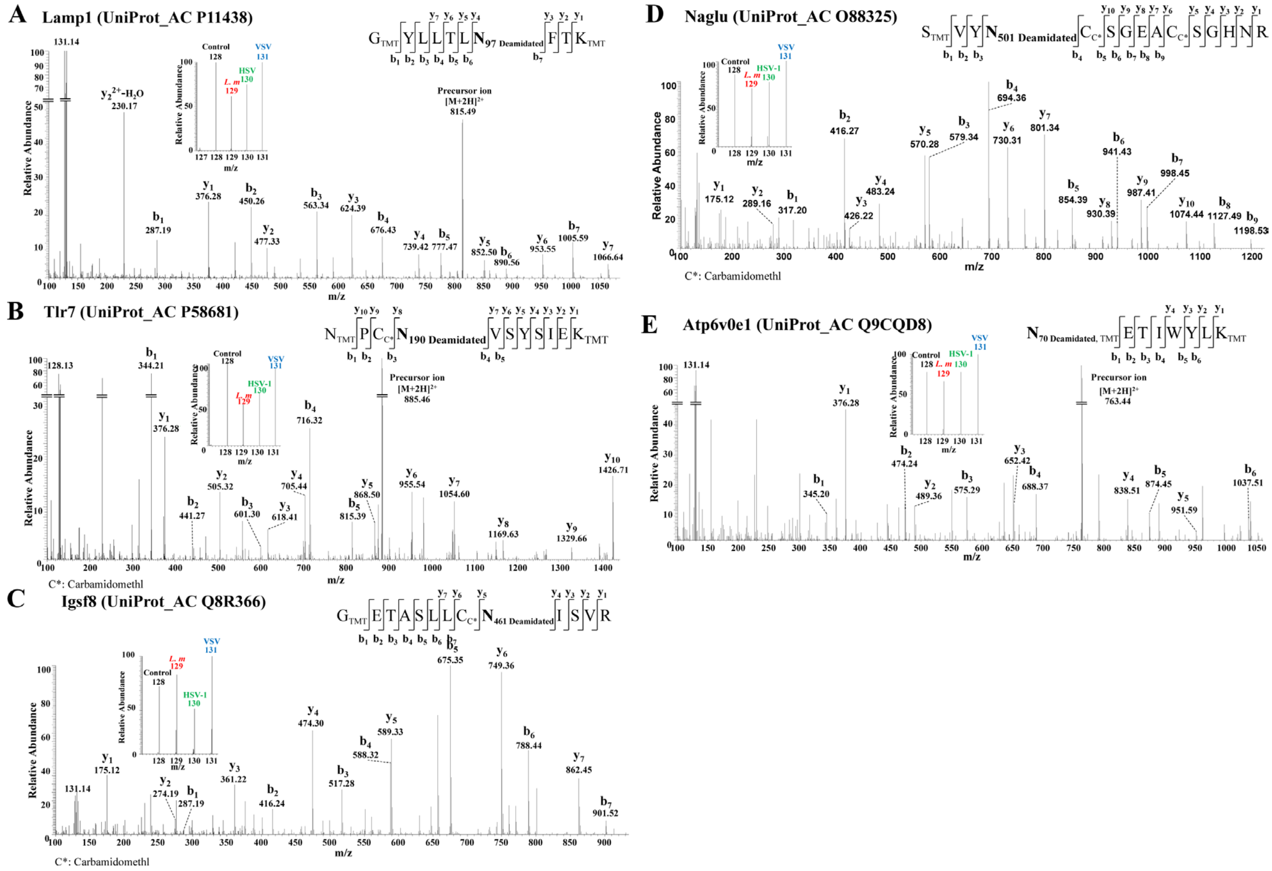

Supplementary Figure 4: MS/MS data of representative peptides demonstrating the N-linked glycosylation site. A, Reported glycosylation site on Lamp1. B, Predicted glycosylation site by Uniprot on TLR7. C, New potential glycosylation site on glycoprotein Igsf8. D-E, New potential glycosylation site on Naglu and Atp6v0e1.
